# Cost-utility analysis of palivizumab for preventing respiratory syncytial virus in preterm neonates and infants in Colombia

**DOI:** 10.1186/s12879-024-09300-5

**Published:** 2024-04-19

**Authors:** Jaime E. Ordóñez, Victor M. Huertas

**Affiliations:** 1 True Consulting, Medellín, Colombia; 2 Fundación Cardioinfantil, Bogotá, Colombia

**Keywords:** Respiratory syncytial viruses, Premature birth, Bronchopulmonary dysplasia, Congenital heart defects, Palivizumab, Respiratory sounds

## Abstract

**Aim:**

Palivizumab has proven effective in reducing hospitalizations, preventing severe illness, improving health outcomes, and reducing healthcare costs for infants at risk of respiratory syncytial virus (RSV) infection. We aim to assess the value of palivizumab in preventing RSV infection in high-risk infants in Colombia, where RSV poses a significant threat, causing severe respiratory illness and hospitalizations.

**Methods:**

We conducted a decision tree analysis to compare five doses of palivizumab with no palivizumab. The study considered three population groups: preterm neonates (≤ 35 weeks gestational age), infants with bronchopulmonary dysplasia (BPD), and infants with hemodynamically significant congenital heart disease (CHD). We obtained clinical efficacy data from IMpact-RSV and Cardiac Synagis trials, while we derived neonatal hospitalization risks from the SENTINEL-1 study. We based hospitalization and recurrent wheezing management costs on Colombian analyses and validated them by experts. We estimated incremental cost-effectiveness ratios and performed 1,000 Monte Carlo simulations for probabilistic sensitivity analyses.

**Results:**

Palivizumab is a dominant strategy for preventing RSV infection in preterm neonates and infants with BPD and CHD. Its high efficacy (78% in preventing RSV in preterm infants), the substantial risk of illness and hospitalization, and the high costs associated with hospitalization, particularly in neonatal intensive care settings, support this finding. The scatter plots and willingness-to-pay curves align with these results.

**Conclusion:**

Palivizumab is a cost-saving strategy in Colombia, effectively preventing RSV infection in preterm neonates and infants with BPD and CHD by reducing hospitalizations and lowering healthcare costs.

## Introduction

Respiratory syncytial virus (RSV) is a highly contagious respiratory virus that is a common cause of severe lower respiratory tract infections in young children, especially in preterm neonates, infants with bronchopulmonary dysplasia (BPD), and hemodynamically significant congenital heart disease (CHD). RSV is a leading cause of infant hospitalization and can cause severe respiratory illness, including bronchiolitis and pneumonia. Occasionally, RSV can lead to death, particularly in premature infants and those with underlying medical conditions [1]. Palivizumab is a prophylactic measure against severe RSV infection in high-risk children. Palivizumab is a monoclonal antibody administered by injection and works by binding to the RSV virus, preventing it from attaching to and infecting the respiratory tract. Palivizumab can significantly impact the health and well-being of affected children and their families, preventing RSV hospitalizations in preterm neonates and infants with BPD and CHD and recurrent wheezing in childhood [2].

One of the critical clinical impacts of palivizumab is its ability to reduce the number of RSV-related hospitalizations. Several clinical trials have shown that prevention with palivizumab can reduce the risk of hospitalizations by up to 55% in high-risk infants [3]. This reduction in hospitalization is significant in premature infants, who are more susceptible to severe RSV illness and are at increased risk of hospitalization. By reducing the number of RSV-related hospitalizations, palivizumab can lessen the burden on healthcare systems, freeing up hospital beds and other resources for other patients. Moreover, by preventing severe respiratory illness, palivizumab can also reduce the costs associated with hospitalization and other medical interventions [4].

Its ability to reduce the number of RSV-related hospitalizations and prevent severe respiratory illness can improve health outcomes, reduce the burden on healthcare systems, and lower healthcare costs. As such, palivizumab is a crucial component of RSV prevention strategies, particularly in high-risk infants and young children. Therefore, we aim to estimate the value of palivizumab in preventing RSV infection in preterm neonates and infants with BPD and CHD in Colombia in terms of avoided hospitalizations and costs, as well as the health-related quality of life of these patients.

## Methods

The population of this economic evaluation is premature neonates with 35 weeks gestational age (wGA) and less and six months of age or younger or 24 months old or younger and a clinical diagnosis of BPD requiring ongoing medical treatment (i.e., supplemental oxygen, steroids, bronchodilators, or diuretics within the past six months) [3]; or ≤ 24 months old with documented hemodynamically significant CHD and an unoperated or partially corrected CHD [5]. The perspective of the analysis is from the third payer, and the annual discount rate is 5% for costs and benefits [6]. The comparators do not use palivizumab versus five doses of palivizumab (the first two 50 mg doses and the remaining 100 mg doses), and the model time horizon is six years.

**Fig. 1:**
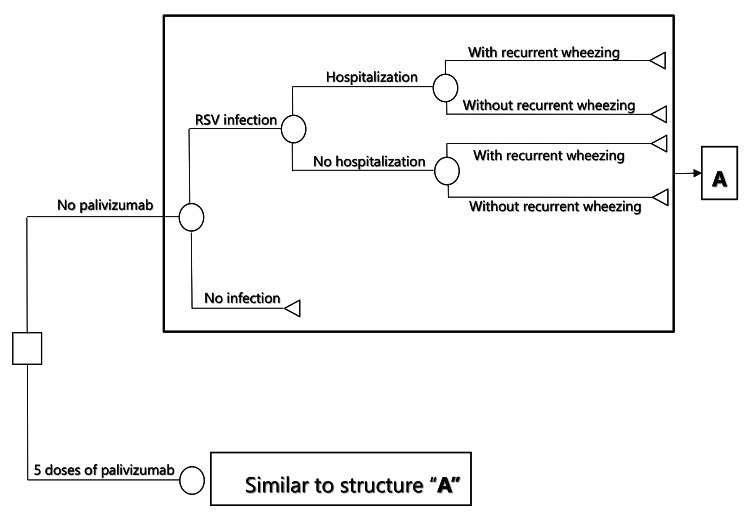
Decision tree model structure with two health states for prophylaxis with palivizumab to prevent the respiratory syncytial virus in children

Clinical outcomes are the rate of RSV-related hospitalizations avoided [3, 5] and the rate of relapses avoided for recurrent wheezing. The costs of the model are in US dollars (USD) for the year 2022, the exchange rate is COP 4,800 per USD 1, and the willingness to pay threshold is one gross domestic product (GDP) per capita in Colombia: $ 6,161 [7]. We did a decision tree model structure with two health states: RSV infection or no infection. Patients with RSV infection can be hospitalized or not hospitalized (outpatient management), and all patients with RSV infection, regardless of their hospitalization state, are likely to have recurrent wheezing or not develop it (Fig. 1).

We took the efficacy of palivizumab in preventing RSV-related hospitalizations in preterm neonates ≤ 35 wGA and infants with BPD from the pivotal study of palivizumab, the IMpact-RSV clinical trial [3]. Similarly, we took the drug’s clinical efficacy in preventing RSV-related hospitalizations in infants with CHD from the Cardiac Synagis clinical trial [5]. In addition, we took the probability of RSV-related hospitalization in preterm neonates ≤ 35 wGA without immune-prophylaxis from the SENTINEL1 study [8]. Finally, we took the distribution of hospitalization services in general wards (47.1%) or intensive care units (ICUs) (52.9%) from a prospective multicentric observational study carried out in six Colombian cities for one year, which included more than 700 infants with RSV-related hospitalization [9].

We took the frequency of RSV-related hospitalizations from a study of hospitalization costs of neonates and infants with RSV infection in Colombia [10]. The risk of developing RSV-related wheezing was taken from a six-year follow-up study of infants with RSV infection [11]. We took the healthcare cost of each RSV-related hospitalization in preterm neonates and infants with BPD from a survey of direct medical costs of RSV-related bronchiolitis hospitalizations in Colombia that included more than 80 infants [12].

We adjusted the cost of each RSV-related hospitalization in infants with CHD based on a cost study in this population. The hospital stay is nearly twice that of infants with RSV-related hospitalizations without CHD [13]. We validated this result with pediatric cardiologists in Colombia. In addition, the weighted mean cost of each wheezing exacerbation (weighted from outpatient care, emergency room (ER), or hospitalization) and the annual relapse rate were taken from a cost study in Colombia’s preschool children with viral-triggered wheeze [14]. Finally, we took inpatient service-adjusted Quality Adjusted Life Years (QALYs) from a study that estimated the quality of life lost due to RSV [15].

We did not find data on the disutility generated by wheezing, so we homologated them to those caused by asthma [16] and validated them with a group of neonatologists. We performed the heterogeneity analysis by estimating costs and outcomes independently for preterm neonates, infants with BPD, and infants with CHD. The model assumes that there are no deaths from RSV infection in neonates with RSV-related hospitalizations since we did not find data on the specific mortality rate from RSV in Colombia. In addition, we performed the cost-utility analysis modeling a cohort of 1000 patients with palivizumab vs. 1000 patients without palivizumab in each group in which the heterogeneity analysis was.

We carried out 1000 Monte Carlo simulations to characterize the uncertainty and estimated the maximum net benefit (MNB) through the willingness to pay curves. Neonatologists and pediatric cardiologists from Colombia participated in constructing the model and validating the inputs.

## Results

Table 1 shows the epidemiological and clinical efficacy parameters of the economic model. Table 2 shows the healthcare costs of each hospital event for patients with RSV-related hospitalizations and the costs of each exacerbation of wheezing. Table 3 shows the QALYs of neonates and infants with RSV infection adjusted for the level of care and the disutilities generated by wheezing. Finally, Tables 4 and 5, and 6 show the discounted total direct costs and QALYs of palivizumab versus placebo in preterm neonates ≤ 35 wGA, in infants with BPD, and infants with CHD in Colombia, respectively.

**Table 1:** Epidemiological parameters of preterm neonates and infants with BPD or CHD, and clinical efficacy of palivizumab in preventing RSV-related hospitalizations

Parameter	Value	Ref
Probability of hospitalization in preterm neonates with RSV infection without palivizumab.	34.9%	[8]
Probability of hospitalization in infants with BPD with RSV infection without palivizumab.	25.6%	[3]
Probability of hospitalization in infants with CHD with RSV infection without palivizumab.	19.4%	[5]
Annual frequency of hospitalizations in neonates and infants with RSV infection.	2.52	[10]
Efficacy of palivizumab to prevent RSV-related hospitalizations in preterm neonates ≤ 35 wGA	78.1% (95% CI: 24.0 − 41.3%)	[3]
Efficacy of palivizumab to prevent RSV-related hospitalizations in infants with BPD.	38.5% (95% CI: 4.95 − 60.2%)	[3]
Efficacy of palivizumab to prevent RSV-related hospitalizations in infants with CHD.	45.3% (95% CI: 18.1 − 63.5%)	[5]
Probability of developing wheezing in neonates and infants with RSV infection.	23.9% (95% CI: 15.9 − 35.8%)	[11, 17]
Annual frequency of relapses due to wheezing in neonates and infants with RSV infection.	4.63 (95% CI: 3.70–5.78)	[14]

**Table 2:** Direct healthcare costs of hospitalizations in patients with RSV infection and weighted mean cost of each wheezing exacerbation in Colombia, 2022

Parameter	Direct healthcare costs (USD)	Ref
Cost of each RSV-related hospitalization in preterm neonates and infants with BPD.	$ 2,076	[12]
Cost of each RSV-related hospitalization in infants with CHD.	$ 3,772	[12, 13]
The weighted mean cost of each wheezing exacerbation (outpatient, ER, or hospitalization).	$ 174	[14]

**Table 3:** QALYs in preterm neonates and infants with RSV adjusted for level of care and disutilities caused by recurrent wheezing in infancy

Clinical parameter	QALYs & disutilities	Ref
RSV without hospitalization.	0.95 (95% CI: 0.76–1.00)	[18, 19]
Hospitalization in the general ward.	0.88 (95% CI: 0.70–1.00)	[15]
Hospitalization in the ICU without acute complications.	0.73 (95% CI: 0.58–0.87)	[15]
Hospitalization in the ICU with acute complications.	0.51 (95% CI: 0.40–0.60)	[15]
Disability due to recurrent wheezing (similar to asthma).	0.087	[16]

**Table 4:** Total discounted direct costs, RSV-related hospitalizations, and QALYs of palivizumab vs. placebo in preterm neonates ≤ 35 wGA in Colombia, 2022

Outcome	No palivizumab	Palivizumab
Total discounted direct costs	$ 4,056,239	$ 2,534,193
RSV-related hospitalizations	349	76
∆ Costs (palivizumab– no palivizumab)	- $ 1,522,046	
∆ QALYs (palivizumab– no palivizumab)	125	

**Table 5:** Total discounted direct costs, RSV-related hospitalizations, and QALYs of palivizumab vs. placebo in infants with BPD, Colombia, 2022

Outcome	No palivizumab	Palivizumab
Total discounted direct costs	$ 2,970,739	$ 2,795,546
RSV-related hospitalizations	256	157
∆ Costs (palivizumab– no palivizumab)	- $ 175,193	
∆ QALYs (palivizumab– no palivizumab)	45	

**Table 6:** Total discounted direct costs, RSV-related hospitalizations, and QALYs of palivizumab vs. placebo in infants with CHD, Colombia, 2022

Outcome	No palivizumab	Palivizumab
Total discounted direct costs	$ 3,921,289	$ 2,811,495
RSV-related hospitalizations	194	106
∆ Costs (palivizumab– no palivizumab)	- $ 1,109,794	
∆ QALYs (palivizumab– no palivizumab)	40	

**Fig. 2:**
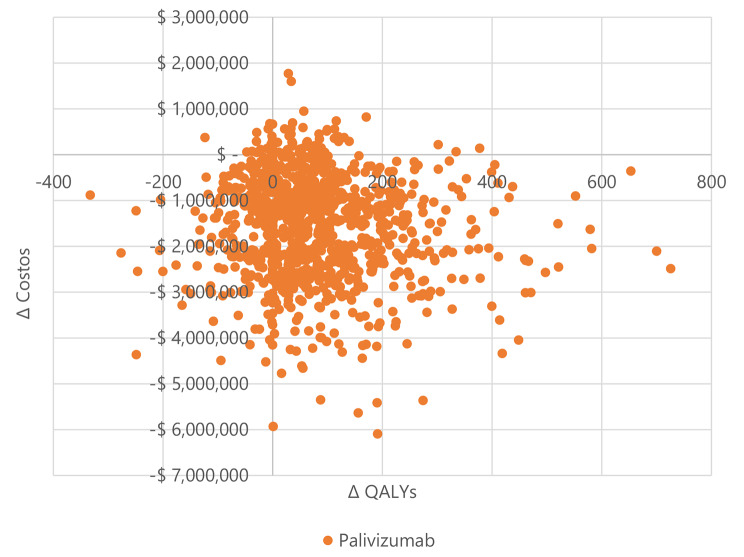
Scatterplot with 1 000 Monte Carlo simulations of the incremental costs and benefits of palivizumab vs. placebo in preterm neonates ≤ 35 wGA, Colombia, 2022

**Fig. 3:**
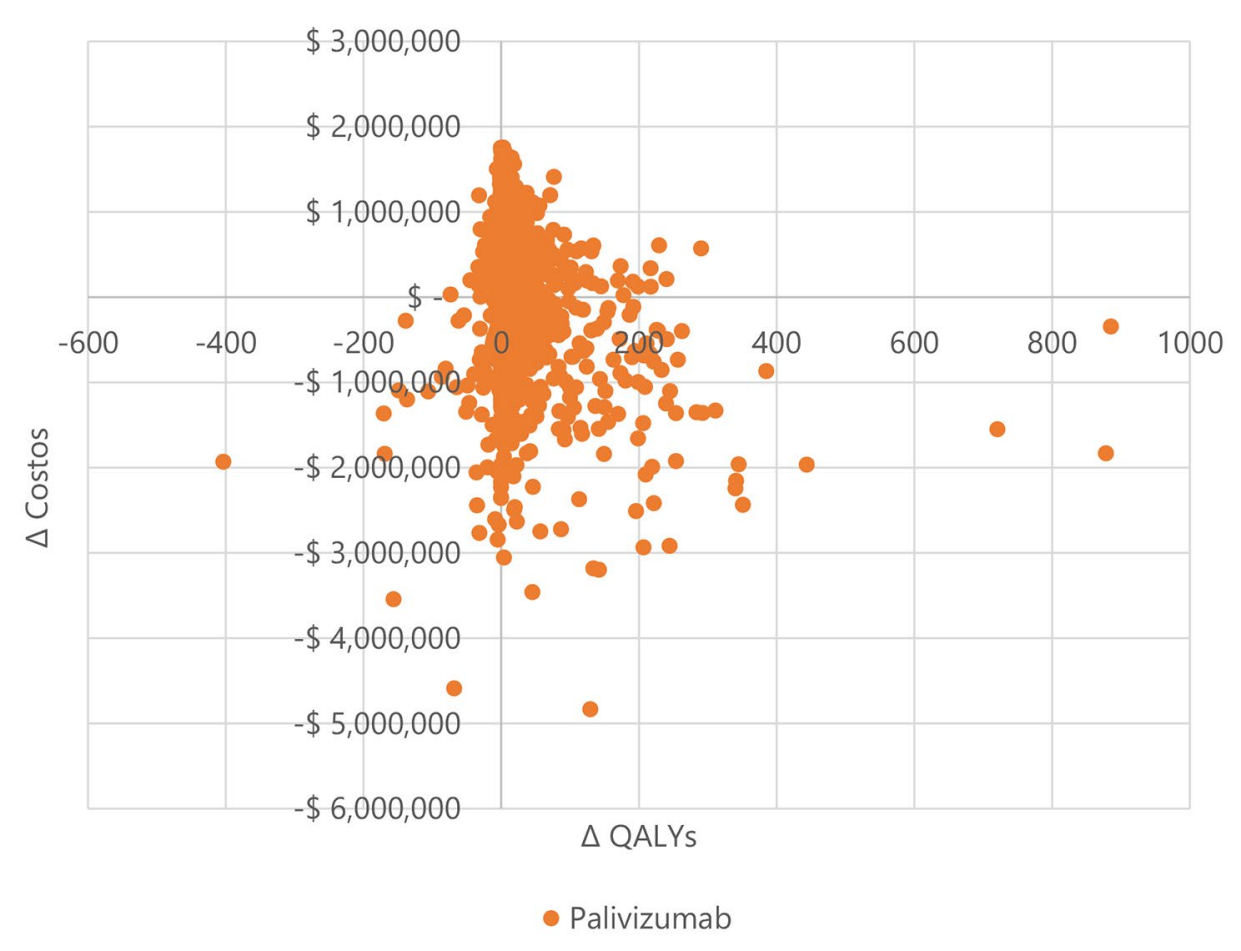
Scatterplot with 1 000 Monte Carlo simulations of the incremental costs and benefits of palivizumab vs. placebo in infants with BPD, Colombia, 2022

**Fig. 4:**
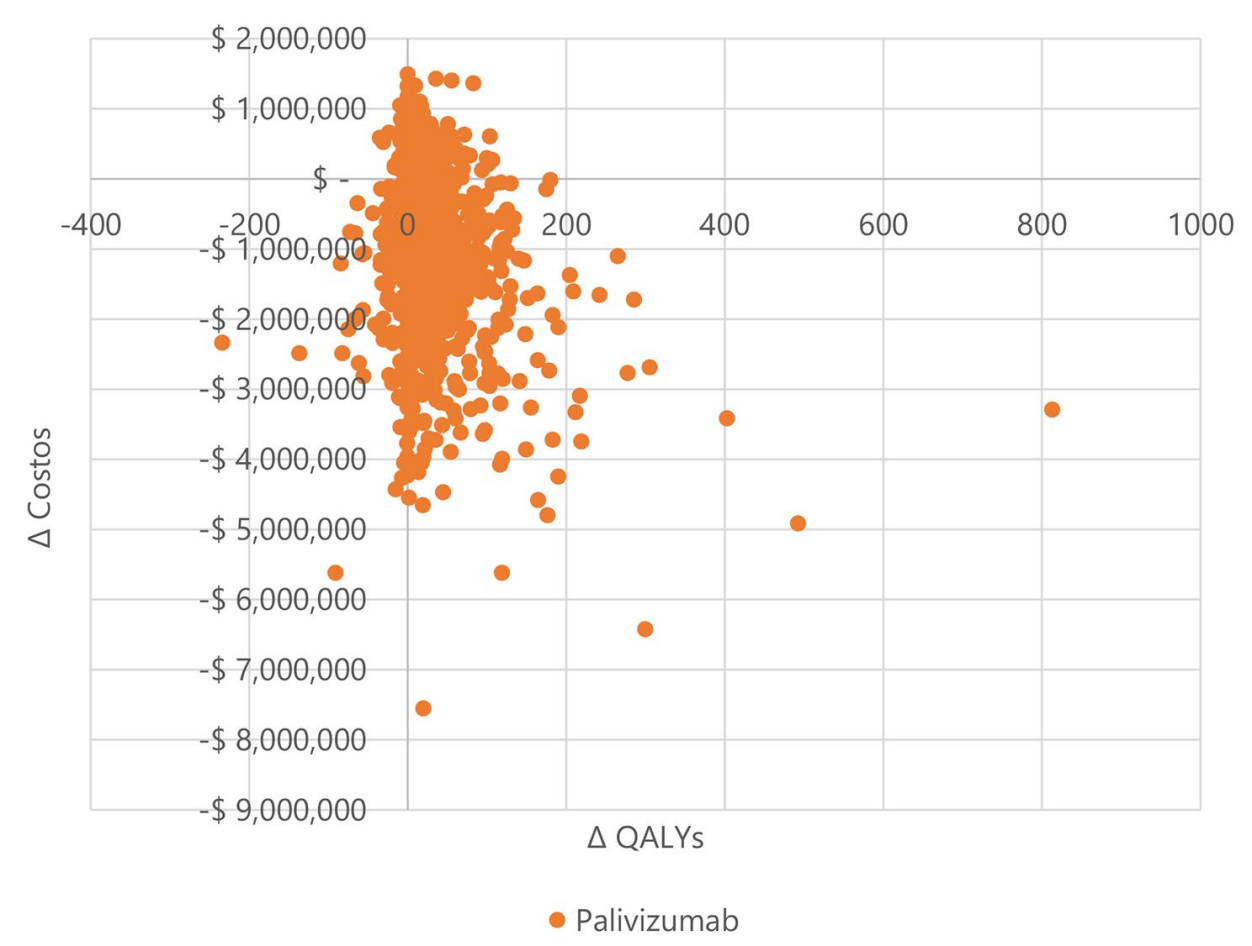
Scatterplot with 1 000 Monte Carlo simulations of the incremental costs and benefits of palivizumab vs. placebo in infants with CHD, Colombia, 2022

The population that receives palivizumab generates lower total costs than those that do not receive it because they have fewer RSV-related hospitalizations due to the drug’s clinical efficacy that prevents them. We observed this result in all population groups: premature neonates and infants with BPD or CHD. Figures 2 and 3, and 4 show the results of the 1,000 Monte Carlo simulations of palivizumab versus placebo in preterm neonates ≤ 35 wGA, in infants with BPD, and infants with CHD in Colombia, respectively. In preterm neonates ≤ 35 wGA (Fig. 2), most simulations are in the southeast quadrant of the cost-effectiveness plane, showing the robustness of the probabilistic results that coincide with the deterministic results.

**Fig. 5:**
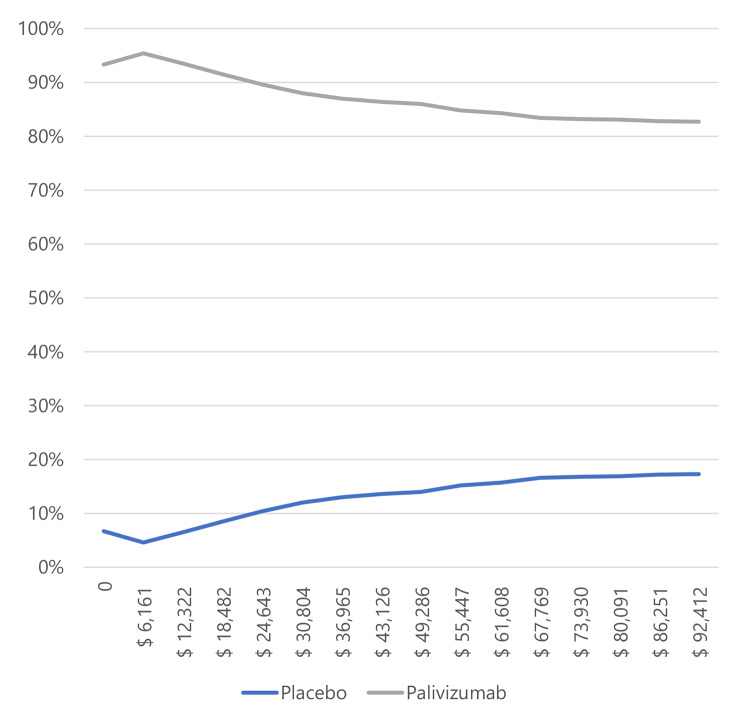
Willingness to pay curves to obtain the Maximum Net Benefit of palivizumab vs. placebo in preterm neonates ≤ 35 wGA in Colombia, 2022

**Fig. 6:**
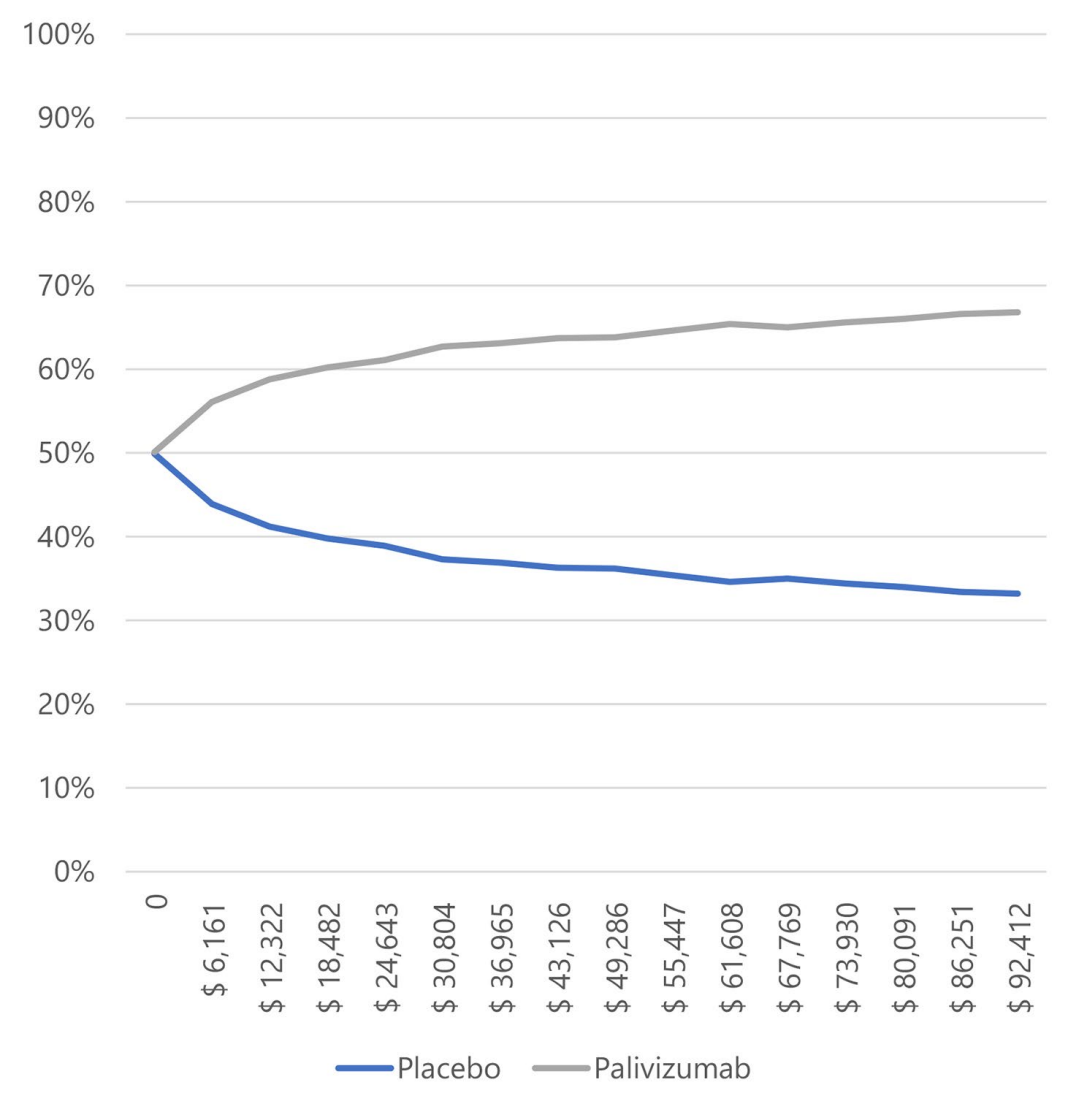
Willingness to pay curves to obtain the Maximum Net Benefit of palivizumab vs. placebo in infants with BPD in Colombia, 2022

**Fig. 7:**
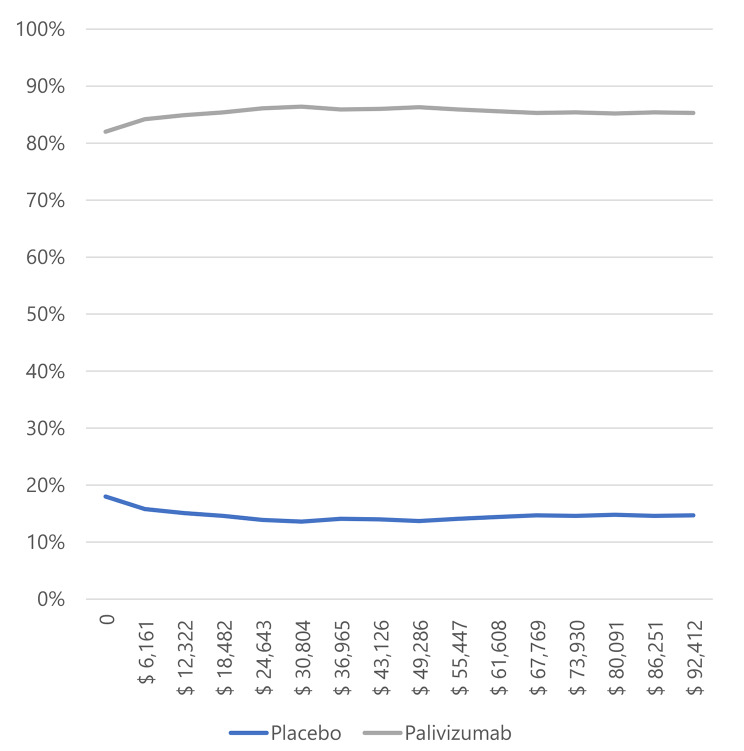
Willingness to pay curves to obtain the Maximum Net Benefit of palivizumab vs. placebo in infants with CHD in Colombia, 2022

In infants with BPD and CHD (Figs. 3 and 4), most simulations are in the cost-effectiveness plane’s right (east) quadrants. It means that the results are cost-effective or dominant, and we observe the scatterplot grouped with very few outliers, indicating that the results are robust. Figures 5 and 6, and 7 show the results of the willingness to pay curves of palivizumab versus placebo in preterm neonates ≤ 35 wGA, in infants with BPD, and infants with CHD in Colombia, respectively. The probability that palivizumab is an effective therapeutic option to prevent RSV infection compared to placebo with a willingness to pay 1 GDP per capita in Colombia is 95.4% in preterm neonates ≤ 35 wGA, 56.1% in infants with BPD, and 84.2% in infants with CHD.

## Discussion

Palivizumab is a cost-saving or dominant strategy to prevent RSV infection in preterm neonates ≤ 35 wGA and infants with BPD or CHD in Colombia. This cost reduction occurs thanks to the clinical efficacy of palivizumab in preventing RSV-related hospitalizations since each hospitalization costs between $ 2,076 and $ 3,772. Similarly, the prevention of wheezing exacerbations improves patients’ quality of life. It saves healthcare costs because the average price of each relapse is $ 174 (a weighted average that includes management in outpatient, ER, or hospitalization).

Since patients have an average of 4.63 (3.70–5.78) relapses per year for at least the first six years of life, this results in savings of $ 4,834 ($ 3,863 - $ 6,034) per patient, maybe $ 174 for a relapse wheezing does not generate a significant economic impact on the Colombian’ health system, but $ 4,834 represents substantial savings, especially in low- and middle-income countries (LMIC). Likewise, the savings generated by avoided RSV-related hospitalizations fluctuate between $176 and $1,522 per patient, results confirmed in the 1,000 Monte Carlo simulations.

For this reason, the willingness to pay curves show that palivizumab has a probability of being an effective option to prevent RSV infection in Colombia, between 56.1% and 94.5%. This variation is because the risk of RSV-related hospitalization in preterm neonates ≤ 35 wGA is higher than in infants with BPD, and the efficacy of palivizumab in preventing RSV infection in preterm neonates is also higher than in infants with BPD.

Although palivizumab generates economic savings and better clinical results than not using it in all populations, the most significant savings are in neonates ≤ 35 wGA. Therefore, it would mean that health systems can offer the most effective clinical benefit and receive the most important economic benefit from applying palivizumab in preterm neonates ≤ 35 wGA. Although the costs of each RSV-related hospitalization of infants with BPD and those of preterm neonates ≤ 35 wGA are similar, the benefit is more significant in preterm neonates. This result is because they have a high risk of hospitalization, and palivizumab efficacy is almost double in preterm neonates than in infants with BPD.

Infants with CHD have a slightly lower risk of RSV-related hospitalization than infants with BPD, and the efficacy of palivizumab is somewhat higher in infants with CHD than in those with BPD (45.3% vs. 38.5%). However, the savings generated to the health system in infants with CHD are more significant than in infants with BPD because the cost of each hospitalization for infants with CHD is higher. It happens because their length of stay (LoS) is usually longer [13], and they typically need more expensive diagnostic tests due to their cardiovascular pathology. As the cost of this population is higher, the savings generated by RSV-related hospitalizations in infants with CHD are also more significant than those generated in infants with BPD.

The conclusions of this economic evaluation are similar to at least 30 other economic evaluations of palivizumab [20–49] since the fabricant brought it to the market. Similarly, several systematic reviews on the cost-effectiveness of palivizumab versus not using it have similar conclusions to this economic evaluation [4, 50, 51]. Therefore, the amount of evidence with similar findings on the cost-effectiveness of palivizumab in preventing RSV infections increases the validity of this economic evaluation. Furthermore, this monoclonal antibody’s cost-effectiveness is mainly due to its high clinical efficacy. Some authors have pointed it out in their meta-analyses [52, 53] based on the multiple published clinical trials evaluating palivizumab’s effectiveness in preventing RSV infections.

Palivizumab has been on the market for a quarter of a century, long enough to demonstrate its high safety, on which several systematic reviews have been published [54–56]. It is essential not only from a clinical point of view but also from an economic point of view since using palivizumab does not generate adverse events that increase healthcare costs. Considering that healthcare costs caused by RSV-related hospitalizations and wheezing relapses are high, it is essential to prevent them from continuing to grow due to adverse events of the medication necessary to prevent it, so this safety contributes to the dominance of palivizumab in Colombia.

This study focuses on preterm neonates with ≤ 35 wGA, like the population in the palivizumab clinical trial (IMpact-RSV) [3]. The largest preterm infant cohort with RSV infection (without immunoprophylaxis) is from the SENTINEL1 study, involving 1,360 infants 29–35 wGA [8]. The neonates were divided into three groups by gestational age: 29–32 wGA, 33–34 wGA, and 35 wGA. They assessed the risk of RSV-related ICU hospitalization and the need for invasive mechanical ventilation (IMV) in each group. The SENTINEL1 study was conducted at 46 sites across the United States [8].

Anderson et al. included 441 neonates at 29–32 wGA, 557 at 33–34 wGA, and 362 at 35 wGA, with ICU admission rates of 48.2%, 45.7%, and 40.0%, respectively. Among those in ICUs, 46.0%, 44.8%, and 61.6% required IMV for 29–32 wGA, 33–34 wGA, and 35 wGA, respectively [8]. These data suggest a similar risk of IMV in RSV-related hospitalizations for neonates aged 29–35 wGA.

The SENTINEL study reported an ICU length of stay (LoS) with a mean of 9 days (SD 9) and an interquartile range (IQR) of 3–13 days for 29–32 wGA, nine days (SD 10) and IQR 3–13 days for 33–34 wGA, and seven days (SD 8) and IQR 3–10 days for 35 wGA. Despite 35-wGA neonates having a 2-day shorter mean LoS, there is overlap in the IQRs, indicating similar ICU stays across these groups.

Concerns regarding antimicrobial resistance (AMR) were raised by a recent Cochrane review on antibiotic use in bronchiolitis [57], and the WHO highlighted AMR’s public health and economic impact [58]. A global AMR study revealed 1.27 million bacterial AMR-associated deaths out of 4.95 million worldwide in 2019 [59]. A UK government review warned that without action, AMR deaths could reach 10 million by 2050, surpassing cancer [60]. The WHO’s global action plan on AMR includes objectives to reduce infection rates and invest in new interventions. As passive immunization, palivizumab lowers RSV-related hospitalizations and antibiotic use, aligning with these objectives and potentially saving costs for the Colombian health system [61].

The main strengths of this economic evaluation are its heterogeneity analysis, which makes it possible to show the results separately for each of the populations of interest, as well as having data on health care costs for this population in Colombia and its distribution in the different care levels. In this way, it was possible to identify that palivizumab generates savings in all interest groups. Although the population with the most savings generated are preterm neonates 29–35 wGA, it is also clear that its use in infants with BPD generates savings for the health system and allows beneficial clinical results.

Although the economic evaluation results are positive, the authors identified some weaknesses that we could resolve in the future with more data to update this study. First, the study assumed no RSV-related mortality because we wanted to focus the analysis on preventing RSV-related hospitalization. However, we must affirm that RSV does cause infant mortality, as evidenced in multiple systematic reviews and disease burden studies [62–64]. By not including the RSV mortality rate in infants, the QALYs in the population not receiving palivizumab are higher than they are since more infants would die in the group not receiving palivizumab. Therefore, the ∆ QALYs generated by palivizumab may be greater than those estimated in this study.

This economic evaluation did not calculate the indirect costs due to the lost productivity of these children’s caregivers (usually the mother). Maternity leave in Colombia is 18 weeks (four months) [65], and the time horizon of this economic evaluation is six years. Unfortunately, these children suffer several RSV-related hospitalizations, especially during the first two years of life, and will subsequently be at risk of wheezing relapses, some of which will resolve in the ER. On other occasions, they will require hospitalization. In addition, children need the accompaniment of a caregiver who will incur a series of expenses, including lost productivity due to absenteeism. The model did not consider these costs because the perspective of the analysis is from the third payer.

Finally, the model did not include two types of costs. First, as mentioned before, AMR is a public health problem and economic problem because it leads to more extended stays and higher costs. Since we do not have data from Colombia that indicate the proportion of infants with bronchiolitis who receive antibiotics and the ratio of them who develop AMR, we decided not to include it. In the same way, there are the opportunity costs of not being able to treat infants with other pathologies that are not preventable on time in the ICUs. Second, Colombia is an LMIC; unfortunately, it does not have enough neonatal ICUs to serve the entire population that needs it.

Like any LMIC, Colombia’s health system must prioritize admitting the most severely ill patients to this service. Unfortunately, many patients cannot enter this service, even if needed, because other patients with more severe diseases occupy it. There is not much that the country’s health system can do except to open more neonatal ICUs, but this is not easy due to their high costs. So, a strategy to offer timely health care to the largest possible population is to avoid the admission of these patients by using immunoprophylaxis with palivizumab. In that way, it could reduce the risk of RSV-related hospitalization and thus allow access to other kinds of patients with not immune-preventable diseases, such as RSV.

In conclusion, palivizumab is a cost-saving strategy to prevent RSV-related hospitalizations and relapse wheezing in preterm neonates 29–35 wGA and infants with BPD or CHD in Colombia. Furthermore, its clinical efficacy prevents RSV-related hospitalizations and the treatment of relapse wheezing for six years, which generates high costs for the Colombian health system and significantly improves the health-related quality of life of these children and their families.

## Data Availability

The authors took the data to build this article from secondary sources referenced in the manuscript. Data on the risk of RSV hospitalization, risk of developing relapsing wheezing, and efficacy of palivizumab are in references [3, 5, 8, 10, 11, 14, 17]. Data on healthcare costs for these patients are in references [11–14], and data on QALYs in different health states are in references [15, 16, 18, 19].
